# Acute encephalopathy with biphasic seizures and late reduced diffusion: A case report

**DOI:** 10.1097/MD.0000000000022940

**Published:** 2020-10-23

**Authors:** Yan-li Ma, Kai-li Xu, Guo-hong Chen, Li Wang, Yuan Wang, Zhi-peng Jin

**Affiliations:** a Department of Neurology; b Department of Pediatric Intensive Care Unit, Children's Hospital Affiliated to Zhengzhou University, Henan Children's Hospital, Zhengzhou Children's Hospital, Zhengzhou, China.

**Keywords:** acute encephalopathy, AESD, case report

## Abstract

**Rationale:**

Acute encephalopathy with biphasic seizures and late reduced diffusion (AESD) has been reported almost exclusively in the Japanese population.

**Patient concerns:**

A 17-month-old male patient presented with fever and seizures, and subsequently fell into a coma. On the second day, he recovered consciousness. On the fourth day, he developed complex partial seizures and fell into a coma again. On day 10, the fever and seizures subsided. Head computed tomography on the first day showed no abnormalities. Brain diffusion-weighted images on the fourth day revealed reduced diffusion in the bilateral subcortical white matter.

**Diagnosis:**

A diagnosis of AESD was made.

**Interventions:**

The patient was treated with corticosteroids and intravenous immunoglobulin.

**Outcomes:**

At the 4-month follow-up, the patient was able to walk independently, and the epileptic seizures were well controlled.

**Lessons:**

AESD is a rare entity, and treatment with corticosteroids and intravenous immunoglobulin can lead to a favorable prognosis. Clinicians should be aware of this condition, and clinicoradiological features can suggest the diagnosis.

## 1 Introduction

Acute encephalopathy refers to an acute cerebral dysfunction caused by viral infections, metabolic disorders, hepatic or renal dysfunction, and hypertension. Acute encephalopathy with biphasic seizures and late reduced diffusion (AESD) is the most common subtype of acute encephalopathy, and accounts for ∼30% of all cases.^[1]^

AESD is characterized by a prolonged febrile seizure (usually >30 min) as the initial neurological dysfunction, followed by a cluster of secondary seizures and deterioration of consciousness on days 4 to 6. At the first stage of AESD, magnetic resonance imaging (MRI) findings may be negative, while at the second stage, diffusion-weighted images (DWI) show reduced diffusion involving the subcortical white matter.^[2]^

Until now, AESD has been reported almost exclusively in the Japanese population.^[3–6]^ In the literature, AESD has never been reported in the Chinese population. Herein, we report a rare case of AESD in China.

## 2 Case report

A 17-month-old male patient presented with fever and seizures. On the first day of onset, he developed fever and vomiting. Eighteen hours after the onset, he experienced generalized tonic-clonic seizures (twice every half hour). Each episode lasted for several minutes, and subsequently, the patient fell into a coma. On the second day, his consciousness recovered to lethargy, and no seizures occurred. On the fourth day after onset, the patient developed complex partial seizures, and he fell into a coma again. Intravenous phenobarbital was administrated but provided no benefit. Thus, levetiracetam was prescribed. On day 10, the fever and seizures subsided, while the coma remained. Henceforth, the symptoms did not relapse.

**Figure 1:**
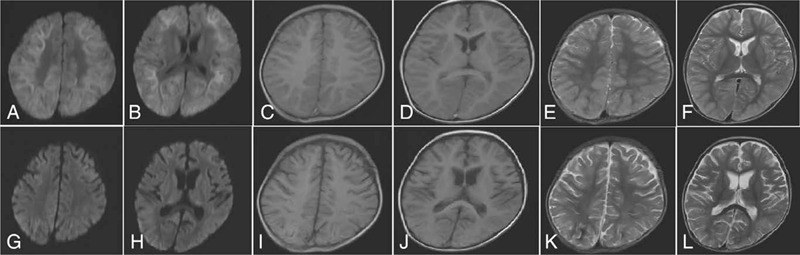
Radiological examination of a 17-month-old male patient with AESD. (A–F) Brain magnetic resonance imaging on the fourth day after onset revealed (A and B) reduced diffusion in the bilateral subcortical white matter on diffusion-weighted images. (C and D) The T1-weighted and (E and F) T2-weighted images were normal. (G and H) Brain MRI on day 14 showed the absence of reduced diffusion on diffusion-weighted images. (I and J) T1-weighted and (K and L) T2-weighted images showed cortical atrophy and focal necrosis.

The previous medical and family histories were unremarkable, and the mental and motor development was normal. On admission, head computed tomography showed no abnormalities. On day 4, brain MRI revealed reduced diffusion in the bilateral subcortical white matter on DWI (Fig. 1A–F). On day 14, brain MRI showed cortical atrophy and focal necrosis, and the reduced diffusion had disappeared (Fig. 1 G and H); T1-weighted and T2-weighted images showed cortical atrophy and focal necrosis (Fig. 1 I–L). Electroencephalogram (EEG) showed diffuse slow waves with a low voltage in the occipital area. At the same time, routine tests of blood, urine, and feces were normal; the C-reactive protein level was elevated (56.64 mg/L). Blood biochemistry showed elevations in the following parameters: alanine aminotransferase (102.0 IU/L); aspartate aminotransferase (134.0 IU/L); hydroxyl butyrate dehydrogenase (1479.0 IU/L); creatine kinase (143.0 IU/L); and creatine kinase-muscle/brain (141.0 IU/L). The renal function, cerebrospinal fluid examination, electrocardiogram, echocardiography, and chest radiography were normal.

The patient was treated with intravenous methylprednisolone (20 mg/kg/d) for 3 days and subsequent oral prednisone for 8 weeks. Additionally, intravenous immunoglobulin (2 g/kg), dehydrant agents, and antiepileptic drugs were administrated. After the 4-month follow-up, the patient was able to walk independently, with poor coordination. The epileptic seizures were well controlled, and oral levetiracetam (30 mg/kg/d) was continued.

## 3 Discussion

The clinical symptoms and radiological characteristics of this patient were consistent with AESD. AESD was originally described by Takanashi in 2006.^[7]^ In Japan, AESD is reported with an estimated incidence of 120 to 200 patients per year.^[8]^ According to the literature, the onset age of AESD ranges from 10 months to 4 years, and most patients have been younger than 2 years. Rarely, there have also been cases of AESD in adults.^[5]^

The definitive pathogenetic mechanism of AESD is still unknown. Recent studies have shown that neurologic inflammation is involved in the pathogenesis of early onset epileptic encephalopathy.^[9]^ Neurotoxic injury of the brain can manifest as delayed abnormalities on neuroimaging. A widely accepted hypothesis attributes AESD to cerebral injury caused by excitotoxicity.^[7]^ Normally, excitatory neurons in the human cortex release excitatory neurotransmitter glutamate, which is absorbed by the surrounding astrocytes from the synaptic space and is metabolized into the relatively harmless complex, glutamine; glutamine returns to the neighboring glutamatergic neurons. When the excessive release of glutamate exceeds the astrocytes’ processing ability, or the ability of astrocytes is insufficient, the accumulation of glutamate in the synaptic space may lead to cell damage. This pathological situation is defined as excitotoxicity. Thus, the source of excitotoxicity in AESD may be disruption of the glutamate-glutamine cycle.

A previous magnetic resonance spectroscopy study showed that the concentration of the glutamate/glutamine complex is elevated in the affected subcortical white matter, which manifested as restricted subcortical diffusion on the third day after the onset of AESD.^[10]^ The glutamate aggregation leads to overexcitation of glutamatergic neurons, which can explain the biphasic seizures. In addition, enlargement of astrocytes induced by glutamate limit the extracellular space, which may be a contributor to the late reduced diffusion.

AESD has consistent clinical manifestations. There are two stages in the acute phase of AESD.^[1,2]^ The first stage is characterized by a prolonged febrile seizure, which usually lasts for >30 min. Consciousness is generally improved on the second day after the episode. At the second stage, the patient develops a cluster of secondary seizures and deterioration of consciousness on days 4 to 6 after the initial onset. It is of note that dyskinesia, dysopia, and dyspnea are also common symptoms in the acute phase.

In the recovery phase of AESD (9 days to 3 months after onset), consciousness is gradually recovered, and epileptic seizures subside. In the sequelae phase, ∼60% of patients suffer from neurological sequelae such as dyskinesia and cognitive deficits, and 30% of patients have epileptic seizures.^[11]^

MRI is usually negative in the first stage of the acute phase of AESD (i.e., the first 2 days after onset). In the second stage of the acute phase (3–9 days after the first episode), the DWI shows decreased diffusion in the subcortical white matter.^[7,12]^ The late reduced diffusion can be diffuse or prominent in the frontal lobes and involve the unilateral or bilateral hemispheres. DWI hyperintensities in the widespread subcortical white matter may be noted, with the characteristic appearance of tree branches. This sign has been termed “bright tree appearance.”^[13]^ In most cases of AESD, the anterior and posterior central sulci are not affected; this is known as “rolandic area avoidance.” In a few cases, T2-weighted imaging or fluid-attenuated inversion recovery images show subcortical linear hyperintensity. In the recovery phase, the hyperintensities on DWI gradually disappear and are replaced by cortical atrophy. In the current case, the radiological features are consistent with typical AESD.

On EEG, in the first stage of the acute phase, AESD is characterized by increased slow waves in the background and a decrease in voltage. In the second stage of the acute phase, an episode pattern and interictal epileptic waves can be noted.^[14]^

Laboratory examinations in AESD are nonspecific. Previous studies have shown that S100 protein and tau protein may be elevated in the first stage of the acute phase, while neuron-specific enolase remains at a normal level.^[15]^

Some scholars have proposed that AESD comprises two subtypes, namely hemiconvulsion-hemiplegia syndrome, and acute infantile encephalopathy, the latter predominantly affecting the frontal lobes.^[16]^ AESD should be differentiated from diagnoses such as complex febrile convulsions; Reye's syndrome; Reye-like syndrome; and metabolic, reversible posterior, toxic, or traumatic encephalopathy. AESD was formerly considered as acute encephalopathy with febrile convulsive status epilepticus (AEFCSE).^[7,17]^ AEFCSE also includes hemiconvulsion-hemiplegia syndrome. Although the number of patients with hemiconvulsion-hemiplegia syndrome has been dramatically reduced because of modern management of status epilepticus, this syndrome is still well known in Europe and reported all over the world.^[18]^ Therefore, more children with AESD can be diagnosed if MRI examination is performed aggressively.

In the acute phase of AESD, the main treatment options are symptomatic and supportive, such as administering anticonvulsant agents, reducing intracranial hypertension, and providing respiratory support therapy. Additionally, there is hypothermia treatment, antiviral therapy, immunotherapy such as methylprednisolone and intravenous immunoglobulin, and plasma exchange. Edaravone has been attempted in order to scavenge free radicals, but the efficacy remains uncertain.^[7]^ Theophylline should be avoided, as it may aggravate the excitotoxicity.^[19]^

The prognosis of AESD is mental retardation, which can range from normal to severe. Compared with other infection-related encephalopathies, the mortality rate of AESD is relatively lower (3–5%). However, the incidence of residual neurological sequelae can reach 60%,^[8]^ and ∼30% of patients have drug-refractory epilepsy.

## Author contributions

MYL and CGH designed most of the investigation, data analysis and wrote the manuscript; XKL, WL, WY, and JZP contributed to interpretation of the data and analyses, revising the manuscript critically for important intellectual content. All the authors have read and approved the manuscript.

**Conceptualization:** Yan-li Ma, Kai-li Xu, Li Wang, Yuan Wang, Zhi-peng Jin.

**Data curation:** Yan-li Ma, Li Wang, Yuan Wang, Zhi-peng Jin.

**Investigation:** Yan-li Ma.

**Methodology:** Yan-li Ma, Kai-li Xu.

**Project administration:** Yan-li Ma.

**Resources:** Guo-hong Chen.

**Supervision:** Kai-li Xu.

**Validation:** Li Wang.

**Visualization:** Yuan Wang.

**Writing – original draft:** Yan-li Ma, Guo-hong Chen, Zhi-peng Jin.

**Writing – review & editing:** Yan-li Ma, Guo-hong Chen, Zhi-peng Jin.
